# Unique crosstalk between platelet and leukocyte counts during treatment for acute coronary syndrome: A retrospective observational study

**DOI:** 10.1097/MD.0000000000032439

**Published:** 2022-12-30

**Authors:** Keisuke Shirasaki, Kosuke Minai, Makoto Kawai, Toshikazu D. Tanaka, Kazuo Ogawa, Yasunori Inoue, Satoshi Morimoto, Tomohisa Nagoshi, Takayuki Ogawa, Kimiaki Komukai, Michihiro Yoshimura

**Affiliations:** a Division of Cardiology, Department of Internal Medicine, The Jikei University School of Medicine, Kashiwa Hospital, Chiba, Japan; b Division of Cardiology, Department of Internal Medicine, The Jikei University School of Medicine, Tokyo, Japan.

## Abstract

In the pathophysiology of acute coronary syndrome (ACS), platelet (PLT) and neutrophil (Neu) crosstalk may be important for activating coagulation and inflammation. It has been speculated that PLTs and Neu may affect each other’s cell counts; however, few studies have investigated this hypothesis. In this study, we measured changes in blood cell counts in 245 patients with ACS during treatment and investigated the mutual effects of each blood cell type. Path diagrams were drawn using structural equation modeling, and temporal changes in the count of each blood cell type and the relevance of these changes were analyzed. Throughout the treatment period, the numbers of all blood cell types (red blood cells [RBCs], leukocytes, and PLTs) were associated with each other before and after treatment. A detailed examination of the different cell types revealed that the PLT count at admission had a significant positive effect on the leukocyte (especially Neu) count after treatment. Conversely, the leukocyte (especially Neu) count at admission had a significant positive effect on the PLT count after treatment. During ACS, PLTs and leukocytes, especially Neu, stimulate each other to increase their numbers. The formation of a PLT-leukocyte complex may increase coagulation activity and inflammation, which can lead to a further increase in the counts of both blood cell types.

## 1. Introduction

Acute thrombus formation in the coronary arteries causes acute coronary syndrome (ACS). Increased coagulability is a systemic problem, not just a problem in the heart.^[1]^ Platelet (PLT) function is generally considered to be central to thrombus formation. However, it is difficult to accurately determine PLT function in clinical practice because the index obtained in routine clinical practice is the number of PLTs. Interestingly, recent reports suggest that the PLT count alone may partially reflect the pathology of ACS.^[2,3]^ In addition, it appears that the thrombotic condition of ACS can be assessed by the PLT-to-lymphocyte ratio (PLR).^[4,5]^ Importantly, both the PLT count and leukocyte count have been shown to be involved in the pathology of ACS.^[6–8]^ The neutrophil-to-lymphocyte ratio (NLR) is closely associated with the pathology of ACS.^[9,10]^ Thus, it is likely that both PLTs and Neu are deeply involved in the thrombotic activity of ACS.

As mentioned above, the number of PLTs and Neus in the blood and the pathophysiology of ACS are thought to be closely related. However, it is unclear whether there is an association between the counts of each blood cell type. To the best of our knowledge, no research has been conducted on this topic in the ACS field. Perhaps activated PLTs and Neus affect each other and increase each other’s counts, increasing thrombotic activity and promoting myocardial ischemia.

Therefore, in this study, we investigated time-dependent changes in blood cell counts, particularly changes in PLTs and Neus, and their relationship during the acute and posttreatment stages of ACS. The degree of interaction was analyzed in detail using appropriate statistical analyses.

## 2. Materials and Methods

### 2.1. Patient population

This study included patients with ACS who required emergency admission to Jikei University Hospital between September 2014 and July 2019. ACS was defined as myocardial infarction (MI) and unstable angina pectoris, as described in detail previously.^[11]^ Two of the following 3 criteria were needed for an MI diagnosis: a history of cardiac chest pain lasting at least 30 minutes; typical electrocardiographic changes; and an increase in the serum creatine kinase level. unstable angina pectoris was diagnosed according to the criteria of the Braunwald classification, without an increase in serum creatine kinase levels. During this period, 301 patients were treated for ACS. The following cases were excluded to determine the natural history of ACS due to organic stenosis or thrombi: Death during hospitalization (1 patient), Coronary spastic angina without significant stenosis or thrombosis (42 patients), Patients who underwent blood transfusion, which might have affected the results of the present study (4 patients), and Patients who underwent coronary artery bypass grafting, which can cause inflammatory reactions due to strong external stimulation (9 patients). Based on these selection criteria, 245 consecutive patients were enrolled in the current study. Of the 245 patients studied, 4 had a history of hematologic disease, but none had active disease. One patient had a history of inflammatory bowel disease that was not active during the study period. Five patients had a history of connective tissue disease and were taking steroids; however, there was no change in the prescription during hospitalization. The Ethics Committee of the Jikei University School of Medicine approved the study protocol (24–355[7121]), and we complied with the routine ethical regulations of our institution. This was a retrospective study, and informed consent was not obtained from any of the patients. Instead of obtaining informed consent from each patient, we posted a notice regarding the study design and contact information in a public location at our institution.

### 2.2. Data collection

Clinical characteristics and biochemical data were collected retrospectively from hospital medical records. Hematological data, including red blood cell (RBC), leukocyte, and PLT counts, were obtained at admission and after treatment. Subpopulations of leukocytes (neutrophil, lymphocyte [Lym], monocyte [Mon], eosinophil [Eos], and basophil [Bas] counts) were also assessed. Blood samples were collected at the time of emergency catheter sheath insertion for all but 1 patient, from whom blood was collected at the end of catheter insertion. When patients were admitted for ACS, they were first given intravenous heparin (100 U/kg) in the emergency room. Aspirin (200 mg) plus prasugrel (20 mg) or clopidogrel (300 mg) were also administered orally according to the appropriate guidelines.^[12]^ The patient was then promptly transferred to the catheterization room, and after sheath insertion, admission blood tests were performed, the activated coagulation time (ACT) was measured, and ACT was measured every 30 minutes to 1 hour. Heparin was administered for 250 to 400 seconds to control ACT. Body mass index was calculated as body weight (kg) divided by height (m^2^). Hypertension, diabetes mellitus, and dyslipidemia were defined as described previously.^[13]^

### 2.3. Statistical analysis

Continuous variables are expressed as the mean ± standard deviation. The correlations between admission PLT and leukocyte counts and discharge PLT and leukocyte counts were examined using separate linear regression analysis. The Wilcoxon signed-rank test was used to compare the degree of inflammation and body temperature at admission and at discharge. All data were statistically analyzed using SPSS software (version 25.0; SPSS Inc., Chicago, IL). Statistical significance was set at a *P* value < .05, indicating statistical significance. A path model based on structural equation modeling was used to investigate the relationships between clinical factors in the study population and to survey the probable relationships among RBC, leukocyte subpopulation (neutrophils, Lym, Mon, Eos, and Bas), and PLT counts. Path analysis was performed using IBM SPSS AMOS software (version 25, Amos Development Corporation, Meadville, PA). We previously described how to write a path model.^[14]^ For every regression, the total variance in the dependent variable is theorized to be caused by either independent variables that are included in the model or by extraneous variables (e).^[15,16]^ The structural equation models that were obtained were tested and confirmed; a *P* value < .05 indicated statistical significance. In addition, we applied Bayesian estimation to structural equation modeling using a program embedded in the IBM SPSS AMOS software program (version 25.0; Amos Development Corporation). The frequency polygon is described using the marginal posterior distributions of the estimates. A selected 2-dimensional contour line was used in this study because it could be easily visualized. The credible region (CI) is conceptually similar to a bivariate confidence region that is familiar to most data analysts acquainted with classic statistical inference methods.

## 3. Results

### 3.1. Characteristics of the study participants

The clinical characteristics of the 245 patients are presented in Tables 1 and 2. At discharge, none of the patients received a heparin drip infusion. The percentage of patients taking antiplatelet medications was 100% for aspirin, 38.8% for clopidogrel, and 58.3% for prasugrel, and dual antiplatelet therapy was administered to 97.1% of patients. To compare the degree of inflammation, we compared body temperatures at admission and at discharge. The average body temperatures at admission and at discharge were 36.3 ± 0.8 and 36.3°C ± 0.4°C (median ± interquartile range), and no statistically significant difference was observed. (*P* = .89, Wilcoxon signed-rank test).

**Table 1:** Clinical characteristics.

Characteristic (N = 245)	Mean ± SD, number (%)
Age	61.7 ± 12.1
Male sex (%)	218 (89.0)
Height (cm)	168.2 ± 8.0
BMI (kg/m^2^)	25.2 ± 4.0
Underlying disease	
Hypertension (%)	163 (66.5)
Dyslipidemia (%)	183 (74.7)
Diabetes mellitus (%)	81 (33.1)
Atrial fibrillation (%)	9 (3.7)
Prior MI (%)	26 (10.6)
Prior PCI (%)	33 (13.5)
Prior CABG (%)	4 (1.6)
History of heart failure (%)	6 (2.4)
History of stroke (%)	13 (5.3)
Smoking history	
Current smoker (%)	82 (33.4)
Past smoker (%)	89 (36.3)
Never smoked (%)	74 (30.2)
Medication	
Calcium channel blockers (%)	76 (31.0)
Beta blockers (%)	39 (16.0)
ACE inhibitors (%)	13 (5.3)
ARBs (%)	57 (23.3)
Nitrates (%)	14 (5.7)
Nicorandil (%)	10 (4.0)
Statins (%)	58 (23.7)
Oral antidiabetic agents (%)	46 (18.8)
Insulin (%)	7 (2.9)
Aspirin (%)	53 (21.6)
Clopidogrel (%)	16 (6.5)
Prasugrel (%)	6 (2.4)
Cilostazol (%)	6 (2.4)
Ticlopidine (%)	1 (0.4)
SAPT (%)	40 (16.3)
DAPT (%)	19 (7.8)
Warfarin (%)	1 (0.4)
Rivaroxaban (%)	2 (0.8)
Apixaban (%)	1 (0.4)
Edoxaban (%)	1 (0.4)
Predonine (%)	5 (2.0)
Diagnosis	
STEMI (%)	149 (60.8)
NSTEMI (%)	48 (19.6)
UA (%)	48 (19.6)
Emergent PCI (%)	236 (96.3)
Received PCI (%)	243 (99.1)
Length of hospital stay (d)	9.8 ± 6.4
Length of hospital stay for STEMI (d)	12.4 ± 6.1
Length of hospital stay for NSTEMI (d)	7.4 ± 4.8
Length of hospital stay for UA (d)	4.0 ± 3.0

ACE = angiotensin converting enzyme, ARBs = angiotensin II type I receptor blockers, BMI = body mass index, CABG = coronary artery bypass grafting, DAPT = dual antiplatelet therapy, MI = myocardial infarction, NSTEMI = non-ST elevation myocardial infarction, PCI = percutaneous coronary intervention, SAPT = single antiplatelet therapy, STEMI = ST elevation myocardial infarction, UA = unstable angina.

**Table 2:** Clinical characteristics.

Characteristic (N = 245)	Mean ± SD (%)	Mean ± SD (%)
	Admission	Discharge
BNP (pg/mL)	95.1 ± 176.1	118.9 ± 162.0
Cr (mg/dL)	0.96 ± 1.1	1.0 ± 1.2
RBC (×10^3^/μL)	4.6 ± 0.5	4.4 ± 0.6
Leukocyte (×10^3^/μL)	9.1 ± 3.1	7.0 ± 2.0
Neu (×10^3^/μl)	6.6 ± 3.0	4.6 ± 1.8
Lym (×10^3^/μL)	1.9 ± 0.9	1.7 ± 0.6
Mon (×10^3^/μL)	0.4 ± 0.2	0.5 ± 0.2
Eos (×10^3^/μL)	0.1 ± 0.1	0.3 ± 0.2
Bas (×10^3^/μL)	0.0 ± 0.0	0.0 ± 0.0
PLT (×10^3^/μL)	218.7 ± 51.4	259.2 ± 79.5
PLR	138.2 ± 67.4	167.3 ± 76.3
NLR	4.4 ± 3.1	3.0 ± 1.8

Bas = basophil, BNP = B-type natriuretic peptide, Cr = creatinine, Eos = eosinophil, Lym = lymphocyte, Mon = monocyte, Neu = neutrophil, NLR = neutrophil-to-lymphocyte ratio, PLR = platelet-to-lymphocyte ratio, PLT = platelet, RBC = red blood cell.

### 3.2. Univariate analysis

**Figure 1.**
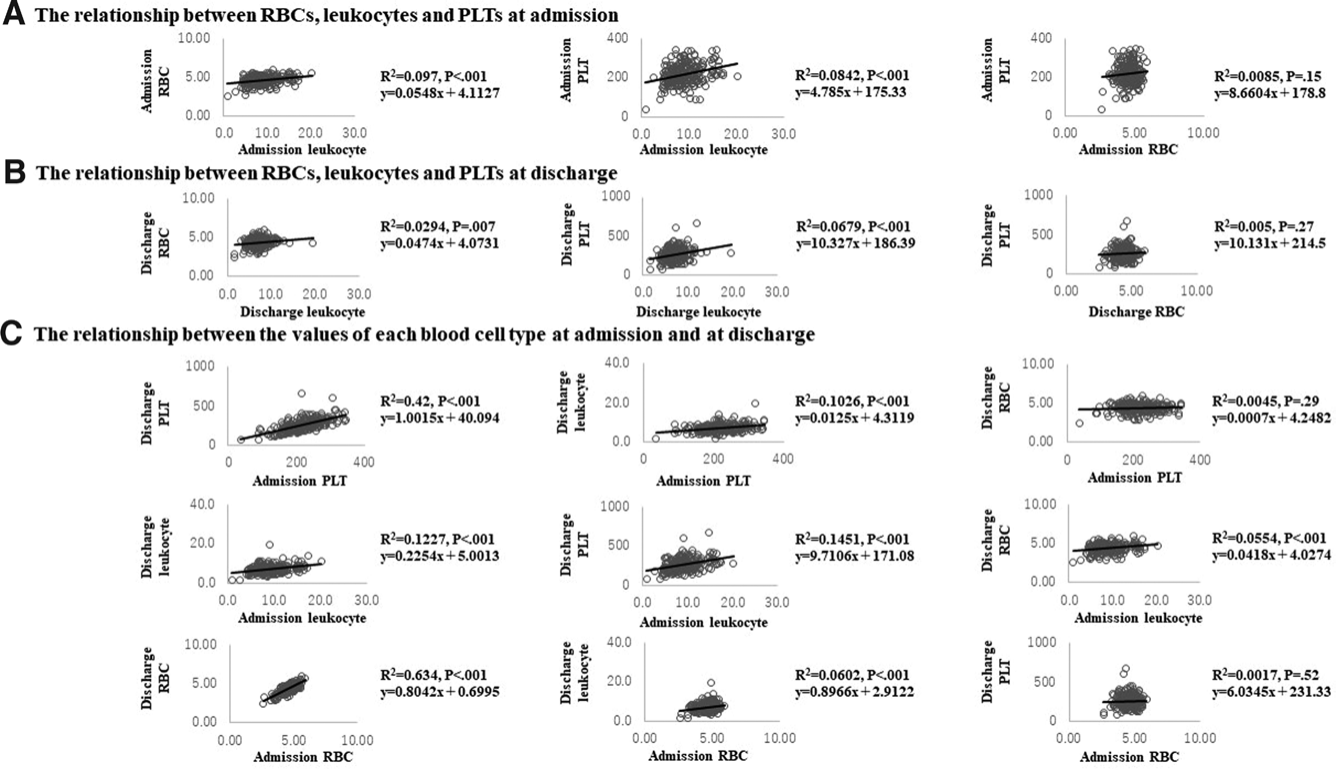
**Univariate analysis.** (a) The relationships between RBC, leukocyte and PLT counts at admission (3 figures). (b) The relationships between RBC, leukocyte and PLT counts at discharge (3 figures). (c) The relationships between the counts of each blood cell type at admission and at discharge (9 figures). PLT = platelet, RBC = red blood cell.

A simple regression analysis revealed the following (Fig. 1). First, at admission, leukocyte and RBC counts (*P* < .001) and leukocyte and PLT counts were correlated (*P* < .001), but RBC and PLT counts were not (*P* = .15). The results obtained at the time of discharge were similar. That is, there was a correlation between leukocyte and RBC counts (*P* = .007) and between leukocyte and PLT counts (*P* < .001) but not between RBC and PLT counts (*P* = .27). In a comparison between admission and discharge. The same blood cell types were correlated at both admission and at discharge (*P* < .001). Regarding the relationship with other blood cells, the PLT counts at admission were correlated with the leukocyte counts at discharge (*P* < .001), and the leukocyte counts at admission were correlated with the PLT counts at discharge (*P* < .001) and with the RBC counts at discharge (*P* < .001). The RBC counts at admission were correlated with the leukocyte counts at discharge (*P* < .001) but not with the PLT counts at discharge (*P* = .52).

### 3.3. Concept of proposed path model (A)

To eliminate confounding biases and clarify the contributions of a RBC counts, leukocyte counts, and PLT counts at admission to RBC counts, leukocyte counts, and PLT counts at discharge more directly, path models based on structural equation modeling were proposed. A theoretical path model was created by positioning the RBC count, leukocyte count, and PLT count at admission in parallel considering the correlations among these 3 factors. The association between these 2 factors is indicated by the 2-way arrow. The paths between variables are drawn from independent to dependent variables, with directional arrows for each regression model.

### 3.4. Results of path model (A)

**Figure 2.**
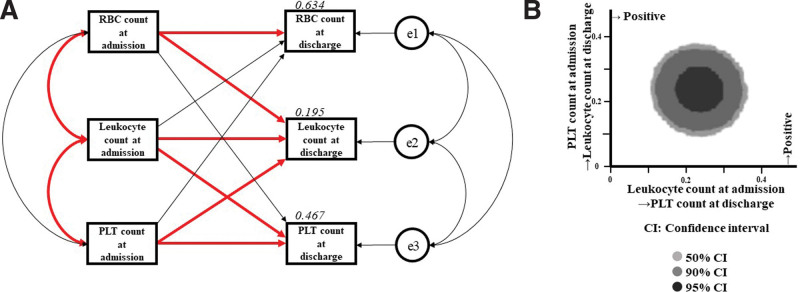
**a (Left): Path model [A] based on structural equation modeling.** An explanatory drawing of the possible cascade from RBC, leukocyte and PLT counts at admission to RBC, leukocyte and PLT counts at discharge. Each path has a coefficient showing the standardized coefficient of regressing an independent variable on a dependent variable in the relevant path. These variables indicate standardized regression coefficients (direct effect), correlations among exogenous variables [red bold typeface indicates significant values] and squared multiple correlations [narrow italics]. **b (Right): Bayesian estimation in structural equation modeling.** Bivariate marginal posterior distributions are shown to help visualize the relationships among pairs of estimands. PLT = platelet, RBC = red blood cell.

The precise results of path model (A) are shown in Table 3 and Figure 2a. There was a significant association between leukocyte counts and RBC counts at admission (correlation coefficient, β:0.331, *P* < .001) and a significant association between leukocyte counts and PLT counts at admission (correlation coefficient, β = 0.290, *P* < .001); however, there were no associations among the 3 at discharge. There were associations between RBC counts (standardized regression coefficient, β:0.801, *P* < .001), leukocyte counts (standardized regression coefficient, β:0.234, *P* < .001), and PLT counts (standardized regression coefficient, β:0.587, *P* < .001) at admission and discharge. Additionally, there were significant positive correlations between PLT counts at admission and leukocyte counts at discharge (standardized regression coefficient, β:0.239, *P* < .001) and between leukocyte counts at admission and PLT counts at discharge (standardized regression coefficient, β = 0.238, *P* < .001). Furthermore, there was a significant positive correlation between the RBC count at admission and leukocyte count at discharge. However, this relationship was weak (standardized regression coefficient, β = 0.15, *P* = .013).

**Table 3:** The results of path model (A) based on structural equation modeling.

Clinical factor			Estimate	Standard error	Test statistic	*P* value	Standard regression coefficient
RBC count at admission	→	RBC count at discharge	0.809	0.041	19.648	<.001	0.801
	→	Leukocyte count at discharge	0.549	0.221	2.486	.013	0.150
	→	PLT count at discharge	−12.541	7.121	−1.761	.078	−0.087
Leukocyte count at admission	→	RBC count at discharge	−0.002	0.008	−0.309	.76	−0.013
	→	Leukocyte count at discharge	0.151	0.040	3.727	<.001	0.234
	→	PLT count at discharge	6.056	1.304	4.645	<.001	0.238
PLT count at admission	→	RBC count at discharge	0.000	0.000	−0.071	.94	−0.003
	→	Leukocyte count at discharge	0.009	0.002	3.975	<.001	0.239
	→	PLT count at discharge	0.907	0.075	12.026	<.001	0.587
Covariance							Correlation coefficient
RBC count at admission	⇄	Leukocyte count at admission	0.530	0.114	4.645	<.001	0.311
	⇄	PLT count at admission	2.596	1.806	1.437	.15	0.092
Leukocyte count at admission	⇄	PLT count at admission	46.307	10.639	4.353	<.001	0.290
e1	⇄	e2	−0.021	0.038	−0.559	.58	−0.036
	⇄	e3	1.936	1.245	1.554	.12	0.100
e2	⇄	e3	1.673	6.651	0.252	.80	0.016

PLT = platelet, RBC = red blood cell.

### 3.5. Results of Bayesian estimation in structural equation modeling

The SEM data were analyzed using Bayesian estimation. Bivariate marginal posterior distributions are shown in Figure 2b. This figure shows the influence of leukocyte number at admission on PLT number at discharge (*x*-axis) and the influence of PLT number at admission on leukocyte count at discharge (*y*-axis). All plots are far from the line relative to zero, and the Bayesian estimation clearly shows the effects of both.

### 3.6. Concept of proposed path model (B)

To eliminate confounding biases and clarify the contributions of the RBC counts, neutrophil counts, Lym counts, Mon counts, Eos counts, Bas counts and PLT counts at admission to the RBC counts, neutrophil counts, Lym counts, Mon counts, Eos counts, Bas counts, and PLT counts at discharge more directly, path models based on structural equation modeling were proposed. A theoretical path model was created by positioning the RBC count, neutrophil count, Lym count, Mon count, Eos count, Bas count, and PLT count at admission in parallel considering the correlations between these 7 factors. The association between these 2 factors is indicated by a 2-way arrow. The paths between variables are drawn from independent to dependent variables, with directional arrows for each regression model.

### 3.7. Results of path model (B)

**Figure 3.**
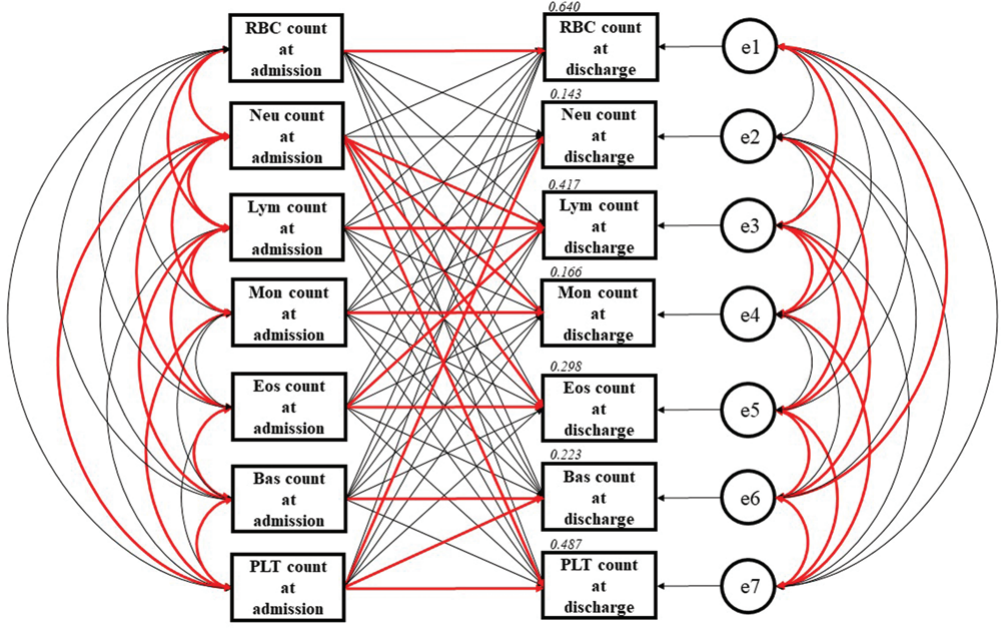
**Path model [B] based on structural equation modeling.** An explanatory drawing of the possible cascade from RBC, Neu, Lym, Mon, Eos, Bas and PLT counts at admission to RBC, Neu, Lym, Mon, Eos, Bas and PLT counts at discharge. Each path has a coefficient showing the standardized coefficient of regressing an independent variable on a dependent variable in the relevant path. These variables indicate standardized regression coefficients (direct effect), correlations among exogenous variables [red bold typeface indicates significant values] and squared multiple correlations [narrow italics]. Bas = basophil, Eos = eosinophil, Lym = lymphocyte, Mon = monocyte, Neu = neutrophil, PLT = platelet, RBC = red blood cell.

The precise results of path model (B) are presented in Table 4 and Figure 3. In the acute phase of ACS, neutrophil counts at admission were positively associated with RBC counts (correlation coefficient, β:0.269, *P* < .001) and PLT counts (correlation coefficient, β:0.248, *P* < .001) at admission but negatively associated with Lym counts at admission (correlation coefficient, β: -0.168, *P* = .009). However, these relationships were not observed after ACS treatment. Neutrophil count at discharge was not associated with RBC count (*P* = .27), PLT count (*P* = .77) or Lym count (*P* = .19) at discharge. The pre- and posttreatment counts of all blood cells, other than neutrophils (*P* = .13), were associated with each other (*P* < .001). Regarding the relationships between different blood cell counts, PLT counts at admission had a significant positive effect on neutrophil counts at discharge (standardized regression coefficient, β = 0.178, *P* = .006). Conversely, neutrophil count at admission had a significant positive effect on PLT count at discharge (standardized regression coefficient, β = 0.199, *P* = .001). In addition, the correlation between RBC counts at admission and leukocyte counts at discharge seen in path model (A) disappeared in this analysis in which leukocyte count was considered.

**Table 4:** The results of path model (B) based on structural equation modeling.

Clinical factor			Estimate	Standard error	Test statistic	*P* value	Standard regression coefficient
RBC count at admission	→	RBC count at discharge	0.810	0.042	19.449	<.001	0.802
	→	Neu count at discharge	0.395	0.205	1.928	.05	0.123
	→	Lym count at discharge	0.041	0.054	0.766	.44	0.040
	→	Mon count at discharge	0.030	0.021	1.434	.15	0.090
	→	Eos count at discharge	−.011	0.019	−.565	.57	−0.033
	→	Bas count at discharge	−0.008	0.004	−1.882	.06	−0.114
	→	PLT count at discharge	−9.397	7.126	−1.319	.19	−0.065
Neu count at admission	→	RBC count at discharge	−0.001	0.009	−0.148	.88	−0.008
	→	Neu count at discharge	0.072	0.047	1.533	.13	0.123
	→	Lym count at discharge	0.050	0.012	4.091	<.001	0.271
	→	Mon count at discharge	−0.009	0.005	−2.000	.05	−0.159
	→	Eos count at discharge	0.015	0.004	3.478	<.001	0.253
	→	Bas count at discharge	0.001	0.001	1.200	.23	0.092
	→	PLT count at discharge	5.199	1.623	3.204	.001	0.199
Lym count at admission	→	RBC count at discharge	−0.026	0.029	−.902	.37	−0.041
	→	Neu count at discharge	−0.072	0.144	−0.500	.62	−0.035
	→	Lym count at discharge	0.424	0.038	11.260	<.001	0.645
	→	Mon count at discharge	−0.013	0.015	−0.871	.38	−0.060
	→	Eos count at discharge	−0.014	0.013	−1.043	.30	−0.066
	→	Bas count at discharge	−0.002	0.003	−0.713	.48	−0.047
	→	PLT count at discharge	−7.368	5.016	−1.469	.14	−0.079
Mon count at admission	→	RBC count at discharge	0.057	0.118	0.479	.63	0.023
	→	Neu count at discharge	0.946	0.583	1.623	.10	0.118
	→	Lym count at discharge	−0.201	0.152	−1.320	.19	−0.079
	→	Mon count at discharge	0.343	0.059	5.828	<.001	0.417
	→	Eos count at discharge	−0.017	0.054	−0.319	.75	−0.021
	→	Bas count at discharge	0.010	0.013	0.792	.43	0.055
	→	PLT count at discharge	30.828	20.242	1.523	.13	0.085
Eos count at admission	→	RBC count at discharge	0.289	0.184	1.570	.12	0.073
	→	Neu count at discharge	0.907	0.906	1.002	.32	0.072
	→	Lym count at discharge	−0.627	0.236	−2.652	.008	−0.157
	→	Mon count at discharge	−0.022	0.091	−0.240	.81	−0.017
	→	Eos count at discharge	0.748	0.084	8.948	<.001	0.582
	→	Bas count at discharge	−0.024	0.020	−1.205	.23	−0.082
	→	PLT count at discharge	0.316	31.459	0.10	.99	0.01
Bas count at admission	→	RBC count at discharge	−0.735	0.628	−1.170	.24	−0.051
	→	Neu count at discharge	4.571	3.092	1.478	.14	0.099
	→	Lym count at discharge	−0.022	0.807	−0.028	.98	−0.002
	→	Mon count at discharge	0.612	0.312	1.958	.05	0.130
	→	Eos count at discharge	0.150	0.286	0.526	.60	0.032
	→	Bas count at discharge	0.437	0.067	6.546	<.001	0.419
	→	PLT count at discharge	−39.487	107.409	−0.368	.71	−0.019
PLT count at admission	→	RBC count at discharge	0.000	0.000	−0.073	.94	0.003
	→	Neu count at discharge	0.006	0.002	2.768	.006	0.178
	→	Lym count at discharge	0.001	0.001	1.724	.09	0.092
	→	Mon count at discharge	0.000	0.000	1.377	.17	0.088
	→	Eos count at discharge	0.000	0.000	0.531	.60	0.031
	→	Bas count at discharge	0.000	0.000	2.600	.009	0.160
	→	PLT count at discharge	0.937	0.077	12.156	<.001	0.606
Covariance							Correlation coefficient
PLT count at admission	⇄	Bas count at admission	0.501	0.130	3.851	<.001	0.254
	⇄	Eos count at admission	0.635	0.462	1.373	.17	0.088
	⇄	Mon count at admission	1.684	0.730	2.308	.02	0.149
	⇄	Lym count at admission	5.159	2.807	1.838	.07	0.118
	⇄	Neu count at admission	38.596	10.282	3.754	<.001	0.248
	⇄	RBC count at admission	2.596	1.806	1.437	.15	0.092
Bas count at admission	⇄	Eos count at admission	0.002	0.000	5.708	<.001	0.393
	⇄	Mon count at admission	0.000	0.001	-0.647	.52	−0.041
	⇄	Lym count at admission	0.007	0.002	3.286	.001	0.215
	⇄	Neu count at admission	−0.011	0.008	−1.401	.16	−0.090
	⇄	RBC count at admission	0.003	0.001	1.848	.07	0.119
Eos count at admission	⇄	Mon count at admission	0.001	0.002	0.326	.74	0.021
	⇄	Lym count at admission	0.042	0.008	5.174	<.001	0.351
	⇄	Neu count at admission	−0.145	0.029	−5.022	<.001	−0.340
	⇄	RBC count at admission	0.005	0.005	1.014	.31	0.065
Mon count at admission	⇄	Lym count at admission	0.050	0.012	4.065	<.001	0.270
	⇄	Neu count at admission	0.291	0.047	6.236	<.001	0.435
	⇄	RBC count at admission	0.014	0.008	1.811	.07	0.117
Lym count at admission	⇄	Neu count at admission	−0.434	0.167	−2.595	.009	−0.168
	⇄	RBC count at admission	0.063	0.030	2.106	.04	0.136
Neu count at admission	⇄	RBC count at admission	0.447	0.110	4.053	<.001	0.269
e1	⇄	e2	−0.038	0.035	−1.094	.27	−0.070
	⇄	e3	0.019	0.009	2.026	.04	0.131
	⇄	e4	−0.005	0.004	−1.368	.17	−0.088
	⇄	e5	0.004	0.003	1.306	.19	0.084
	⇄	e6	0.002	0.001	2.353	.02	0.152
	⇄	e7	1.753	1.212	1.447	.15	0.093
e2	⇄	e3	−0.058	0.045	−1.299	.19	−0.083
	⇄	e4	0.153	0.020	7.711	<.001	0.568
	⇄	e5	−0.039	0.016	−2.435	.02	−0.158
	⇄	e6	−0.006	0.004	−1.565	.12	−0.101
	⇄	e7	1.746	5.942	0.294	.77	0.019
e3	⇄	e4	0.010	0.005	2.144	.03	0.139
	⇄	e5	0.011	0.004	2.676	.007	0.174
	⇄	e6	0.003	0.001	3.105	.002	0.203
	⇄	e7	2.732	1.560	1.751	.08	0.113
e4	⇄	e5	0.000	0.002	−0.117	.91	−0.007
	⇄	e6	0.000	0.000	−0.688	.49	−0.044
	⇄	e7	−1.530	0.608	−2.517	.01	−0.163
e5	⇄	e6	0.001	0.000	2.616	.009	0.170
	⇄	e7	1.440	0.556	2.589	.01	0.168
e6	⇄	e7	0.317	0.130	2.444	.02	0.158

Bas = basophil, Eos = eosinophil, Lym = lymphocyte, Mon = monocyte, Neu = neutrophil, PLT = platelet, RBC = red blood cell.

### 3.8. PLR and NLR at admission and at discharge

We calculated the PLR and NLR and determined that the PLR was 138.2 ± 67.4 at admission and 167.3 ± 76.3 at discharge, and the NLR was 4.4 ± 3.1 at admission and 3.0 ± 1.8 at discharge.

## 4. Discussion

In this study, structural equation modeling was used to investigate the direct relationships between the factors described above by eliminating the conjugates between the factors as much as possible.

As a result, it became clear that the numbers of leukocytes, especially neutrophils, and PLTs were significantly influenced by each other during ACS.

In this study, we examined blood cell counts; however, these numbers may also be associated with blood cell activation. Although it is difficult to prove this phenomenon directly, many studies suggest that these cell count ratios (PLR and NLR) are prognostic indicators of ACS.^[4,5,9,10]^ Receiver operating characteristic curve analysis also revealed that high values of these blood cell types correlated with a PLR above 128 and an NLR above 2.6.^[17]^ In fact, the PLRs and NLRs obtained in the current study tended to be higher than these values. Again, the increased numbers of PLTs and neutrophils are meaningful and may be associated with thrombotic activity.

The PLT-leukocyte complex has been well studied.^[18–21]^ In brief, when vascular endothelial cells are damaged, receptors for cell adhesion factors (glycoprotein GPIbα, GPIIb/IIIa, etc) are expressed on the PLT cell membrane. Tissue factor is important for the interaction between PLTs and leukocytes.^[20,22]^ P-selectins are also important in promoting tissue factor expression^[23,24]^ and fibrin formation.^[25,26]^ Subsequently, neutrophil extracellular traps are involved in thrombosis by promoting fibrin deposition and fibrin network formation.^[27,28]^

**Figure 4.**
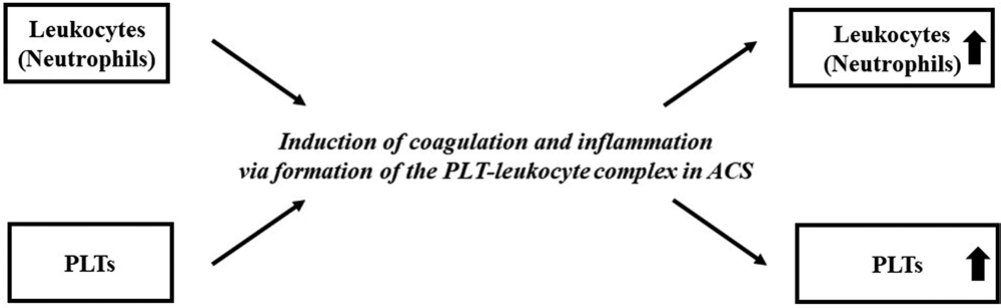
**Conceptual diagram.** This figure shows the possibilities that can be derived from this result. ACS = acute coronary syndrome, PLT = platelet.

As mentioned above, PLTs and leukocytes can affect each other and increase cell numbers during ACS, which is an interesting finding. However, it is unclear why this occurs. It is possible that the PLT-leukocyte complex is also involved. Inflammation is caused by the formation of this complex. Inflammation increases PLT and leukocyte counts.^[29–32]^ In other words, during ACS, the PLT count affects the subsequent leukocyte count, which affects the subsequent PLT count. Crosstalk between PLT and leukocyte counts is a phenomenon that is observed only in ACS and not in non-ACS patients. Figure 4 shows a conceptual diagram of the possibilities derived from this result. At this point, the precise mechanism of crosstalk between PLT and leukocyte numbers remains unclear and is an interesting topic for future research.

Incidentally, the importance of PLT and leukocyte activation has also been highlighted in other conditions, such as stress,^[33]^ HIV,^[34]^ sepsis,^[35]^ ulcerative colitis,^[36]^ and rheumatoid arthritis.^[37]^ Interestingly, these diseases are associated with arteritis and arteriosclerosis, and it is easy to speculate that these conditions may lead to ischemic heart disease. Presumably, the induction of thrombosis and vasculitis by COVID-19 may also be partly related to leukocyte and PLT functions.^[38,39]^

Notably, associations between other types of blood cells were also observed. In this study, Mon were associated with Lym; Bas were associated with Lym, Eos and PLTs; and Eos were associated with Lym. However, the meaning of these relationships remains unclear, and the relationship between the counts and activities of each blood cell type should be investigated in the future.

### 4.1. Study limitations

The limitations of this study are as follows: The number of cases was small. Various drugs other than antiplatelet drugs were used, and the effects could not be eliminated. Regarding structural equation modeling, a path diagram was devised for analysis based on the rich experience of the analysts involved. In general, a path diagram should be as simple as possible and widely accepted. Therefore, in this study, we devised a diagram that considers path symmetry. We also attempted to make the diagram highly intuitive and understandable. However, structural equation modeling is not a method that can be used to examine true causality. These results only show the relationship between blood cell counts, which represents the degree of influence. Further validation using different paths and other statistical methods is required.

## 5. Conclusions

During ACS, PLTs and leukocytes, especially neutrophils, stimulate each other to increase their numbers. The formation of a PLT-leukocyte complex may increase coagulation activity and increases inflammation, which can lead to a further increase in the counts of both types of blood cells.

## Acknowledgments

We thank all trial physicians and nurses at all participating hospitals for their important contributions to this study. We also thank Kumiko Nishiyama for providing assistance with data collection. We thank American Journal Experts (www.aje.com) for English language editing.

## Author contributions

**Conceptualization:** Keisuke Shirasaki, Takayuki Ogawa.

**Data curation:** Kosuke Minai, Makoto Kawai, Toshikazu D. Tanaka, Michihiro Yoshimura.

**Formal analysis:** Kosuke Minai, Makoto Kawai.

**Investigation:** Keisuke Shirasaki, Kosuke Minai, Makoto Kawai, Kazuo Ogawa, Michihiro Yoshimura.

**Methodology:** Keisuke Shirasaki.

**Project administration:** Michihiro Yoshimura.

**Supervision:** Michihiro Yoshimura.

**Validation:** Keisuke Shirasaki, Yasunori Inoue, Satoshi Morimoto, Takayuki Ogawa, Kimiaki Komukai, Michihiro Yoshimura.

**Visualization:** Keisuke Shirasaki, Tomohisa Nagoshi, Michihiro Yoshimura.

**Writing – original draft:** Keisuke Shirasaki.

**Writing – review & editing:** Kosuke Minai, Makoto Kawai, Toshikazu D. Tanaka, Kazuo Ogawa, Yasunori Inoue, Satoshi Morimoto, Tomohisa Nagoshi, Takayuki Ogawa, Kimiaki Komukai, Michihiro Yoshimura.
